# Ability of Antibodies Immobilized on Gold Nanoparticles to Bind Small Antigen Fluorescein

**DOI:** 10.3390/ijms242316967

**Published:** 2023-11-30

**Authors:** Dmitriy V. Sotnikov, Nadezhda A. Byzova, Anatoly V. Zherdev, Boris B. Dzantiev

**Affiliations:** A.N. Bach Institute of Biochemistry, Research Center of Biotechnology of the Russian Academy of Sciences, 119071 Moscow, Russia; nbyzova@inbi.ras.ru (N.A.B.); zherdev@inbi.ras.ru (A.V.Z.); dzantiev@inbi.ras.ru (B.B.D.)

**Keywords:** gold nanoparticles, immunoglobulins, antigen-binding sites, immobilization, conjugates, fluorescein

## Abstract

The analytical applications of antibodies are often associated with their immobilization on different carriers, which is accompanied by a loss of antigen-binding activity for a sufficient proportion of the bound antibodies. In contrast to data on plain carriers, minimal data are available on the properties of antibodies on the surfaces of nanoparticles. Protein antigens have been predominantly investigated, for which space restrictions do not allow them to occupy all active sites of immobilized antibodies. This study considered a low-molecular-weight compound, fluorescein, as an antigen. Spherical gold nanoparticles with five different sizes, two differently charged forms of fluorescein, and three different levels of surface coverage by immobilized antibodies were tested. For gold nanoparticles with diameters from 14 to 35.5 nm with monolayers of immobilized antibodies, the percentage of molecules capable of binding carboxyfluorescein varied from 6% to 17%. The binding of aminofluorescein was more efficient; for gold nanoparticles with an average diameter of 21 nm, the percentage of active binding sites for the immobilized antibodies reached 27% compared with 13% for the carboxyfluorescein case. A fourfold reduction in the coverage of the nanoparticles’ surface compared with that of the monolayer did not lead to reliable changes in the percentage of active binding sites. The obtained data demonstrate that an antigen’s binding to immobilized antibodies is limited even for small antigens and depends on the size of the nanoparticles and the electrostatic repulsion near their surface.

## 1. Introduction

The use of antibodies in immunoassays and immunosensors is often associated with their immobilization on different carriers for the separation of the formed immune complexes from noninteracting compounds and the following generation of registered analytical signals [1,2]. However, immobilized antibodies partially lose their antigen-binding ability due to the violation of their native structure or losing the accessibility to active centers by the antigen molecules. This drawback has been repeatedly reported, and approaches to reduce it have been considered, such as oriented indirect immobilization, the inclusion of bridge structures, etc. [3,4,5,6,7,8]. The inactivation of antibodies via their immobilization on nanoparticles has been much less characterized, although such carriers are used in many widely applied analytical systems and differ significantly from planar carriers [9,10]. The adsorption complexes of antibodies and spherical gold nanoparticles (GNPs) seem to be extremely important for this characterization. They are key compounds of lateral flow test systems that are produced in large quantities to enable simple and rapid testing in medicine, and the use of these systems is extended to food safety, agricultural control, and environmental monitoring [11,12]. Adsorption immobilization is easy to perform and does not involve additional reagents, which is the reason for its wide application. The stored antigen-binding properties of the adsorbed antibodies provide low detection limits and decrease the cost of test systems based owing to these preparations.

The quantitative studies conducted on the reactivity of antibody–GNP complexes have mainly focused on interactions with large protein antigens (peroxidase, C-reactive protein, etc.). To estimate the antigen-binding activity of the antibodies in these complexes, different techniques have been applied, including the fluorescent registration of proteins [13], measurements of catalytic activity using enzyme antigens [14], and the introduction and detection of additional labels [15]. These complementary studies confirmed that a significant proportion of the antibodies do not bind target antigens after their immobilization on GNPs. The percentage of nonactive molecules in the complexes ranged from 17 to 34% [14,15,16] depending on GNP size, the conditions of immobilization, and the features of the antigen. However, in the case of protein antigens, steric restrictions in the interactions between large antigen molecules and antibodies with dense surface immobilization contribute to increased binding. Additionally, steric restrictions on filling all antigen-binding sites occur when the reaction of polyvalent antigens and antibody–GNP conjugates leads to the formation of aggregates. Therefore, special consideration is necessary for low-molecular-weight antigens, for which the indicated above factors are excluded. It is necessary to evaluate the inactivation of immobilized antibody molecules. Earlier, we described the loss of the antigen-binding ability of immobilized antibodies against fluorescein as an example of a small analyte [17]; the found inactivation degree was 88.8%. These data show the manifestation of processes not related to steric restrictions and aggregation. However, the limited number of antibody–GNPs conjugate preparations that were considered in that study did not allow identifying these processes and suggesting their mechanisms.

Based on the above, in this study, the adsorption conjugates of antibodies with GNPs differing in nanoparticle size and antibody:nanoparticle ratios were compared. The use of fluorescein as a low-molecular-weight antigen allows accurate registering of the percentage of its binding via fluorescence measurements. For the correct evaluation of the binding processes, calibration solutions with known concentrations of fluorescein were prepared in the GNP supernatants and compared with the obtained supernatants after immune interactions. The study considered (i) GNPs with different diameters as carriers, (ii) their conjugates with the antibodies immobilized as saturated monolayers and with reduced amounts of antibodies, and (iii) antigen derivatives differing in charge. The established ratios of reactive and inactivated antibodies provided an opportunity to evaluate the consequences of adsorption immobilization and to compare different conjugates as immunoanalytical reagents.

## 2. Results and Discussion

### 2.1. Obtaining and Characterization of Gold Nanoparticles Preparations

**Figure 1:**
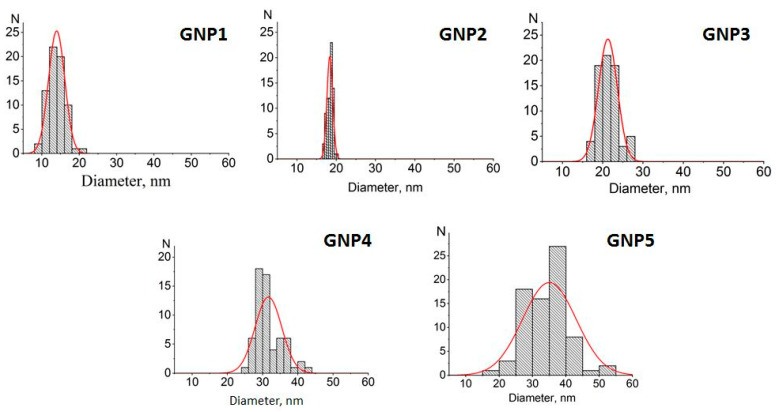
Distributions of diameters for the obtained GNPs (TEM data). Syntheses of GNP1–GNP5 preparations are specified in Section 3.2. Quantities of processed GNP images for each preparation were in the range of 60–90. Columns are histograms of particle size distribution; red curves are approximation of distributions by the Gaussian function.

**Figure A1:**
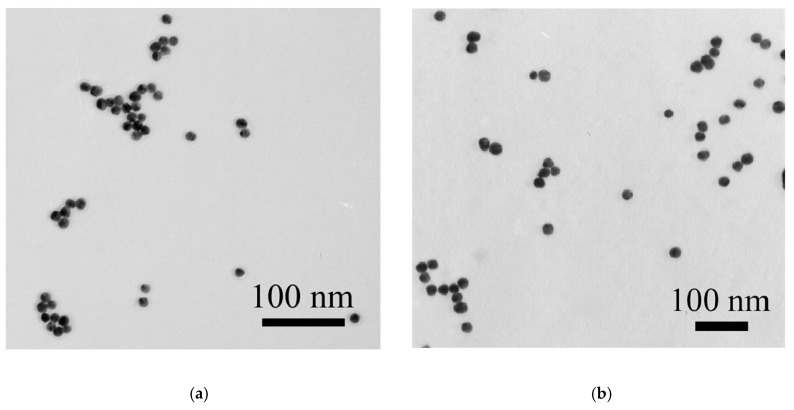

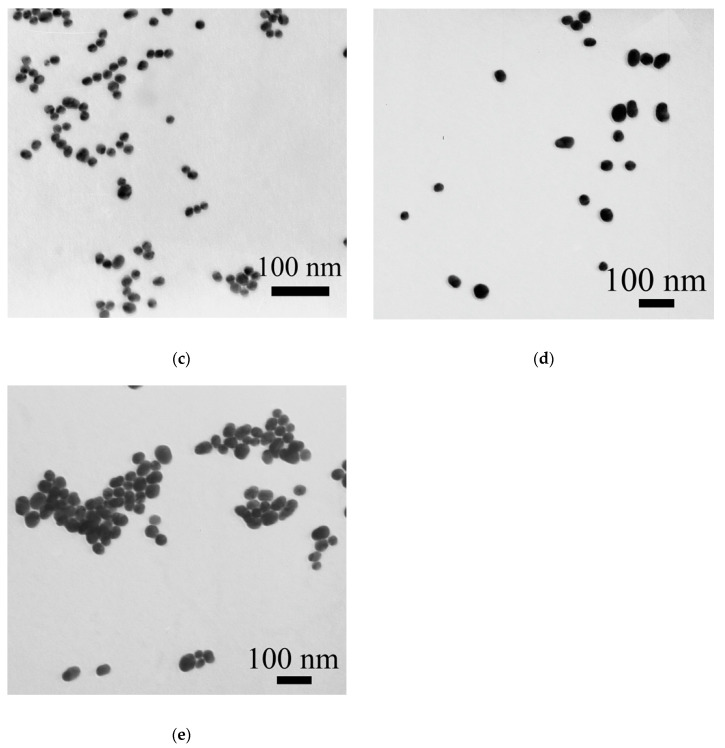
Electron micrographs for GNP1 (**a**), GNP2 (**b**), GNP3 (**c**), GNP4 (**d)**, and GNP5 (**e**) preparations.

Five preparations of GNPs were obtained via the citrate reduction of chloroauric acid under various ratios of reactants. Their micrographs obtained via transmission electron microscopy (TEM) are given in Appendix A (Figure A1), and histograms for the distribution of GNP diameters are depicted in Figure 1. In accordance with the calculated size and shape characteristics (see Table 1), the preparations were further named GNPs with average diameters of 14 nm, 18.5 nm, 21 nm, 31.5 nm, and 35.5 nm. The ellipticity coefficients varied from 1.08 to 1.28, which indicated that the shape of the nanoparticles was close to spherical, especially for the four preparations with smaller diameters.

Based on the earlier established dependence between the average diameter and the peak of the absorption spectrum of spherical gold nanoparticles [16], the GNP preparations were additionally characterized using spectrophotometric data. The found diameters (see Table 1) imperceptibly differed from the microscopic results, from 0.5% (18.5 nm vs. 18.4 nm) to 6.5% (13 nm vs. 13.9 nm), thus confirming the effectiveness of the simple and rapid spectrophotometric characterization of GNPs.

### 2.2. Preparation of Antibody–GNP Conjugates and Determination of Their Composition

Varying the ratio of GNPs and antibodies in the course of the adsorption leads to obtaining products with different compositions and structures. An important difference between these products is whether the antibodies form only one layer or multiple layers on the surface of the particles. Complexes with polylayer immobilization of antibodies on the GNP surfaces have been considered in several studies [18,19,20]. The binding affinity upon the formation of the outer layers is much is lower than the affinity for the first layer, which is why these layers are called soft and hard protein coronas, respectively [21,22,23]. The soft corona can dissociate during the storage and use of conjugates, thereby reducing the yield of detectable complexes, the accuracy, and the reliability of assay results. In addition, the outer layers limit the access of antigen molecules to the first layer of the immobilized antibodies. Therefore, the choice of a monolayer coating of antibodies must be reasonable for analytical purposes [24].

Based on the arguments presented above, the GNP:antibody ratio for their conjugation was chosen in such a way as to be limited to the first layer of immobilized antibodies. For this purpose, we were guided by the fact that the densest arrangement accounts for the occupation of a 25 nm^2^ area on the surface by one IgG molecule. Across the different studies, the measured amounts of IgG adsorbed on GNPs have significantly varied depending on the sorption conditions and the used method. Thus, Driskel’s group, using different methods for determining the concentration of antibodies, studied IgG–GNP conjugates with a diameter of 60 nm [15,25]. They found that 227 to 660 IgG molecules (443.5 on average value) can bind to one particle via physical adsorption. This value corresponds to approximately 25 nm^2^ of GNP surface per one bound IgG molecule; this value was used in our following calculations. To provide the corresponding proportions of reactants for monolayer saturation, their required concentrations were calculated; see Table 2.

The results of the immobilization experiments were characterized through measurements of the composition of the obtained conjugates. As we proposed earlier [16], the fluorescence of tryptophan residues was registered for added IgG solutions and for supernatants with IgG that did not bind to GNPs. The calculated content of the bound antibodies, the percentage of their binding, and the composition of the resulting conjugates are summarized in Table 3.

**Figure A2:**
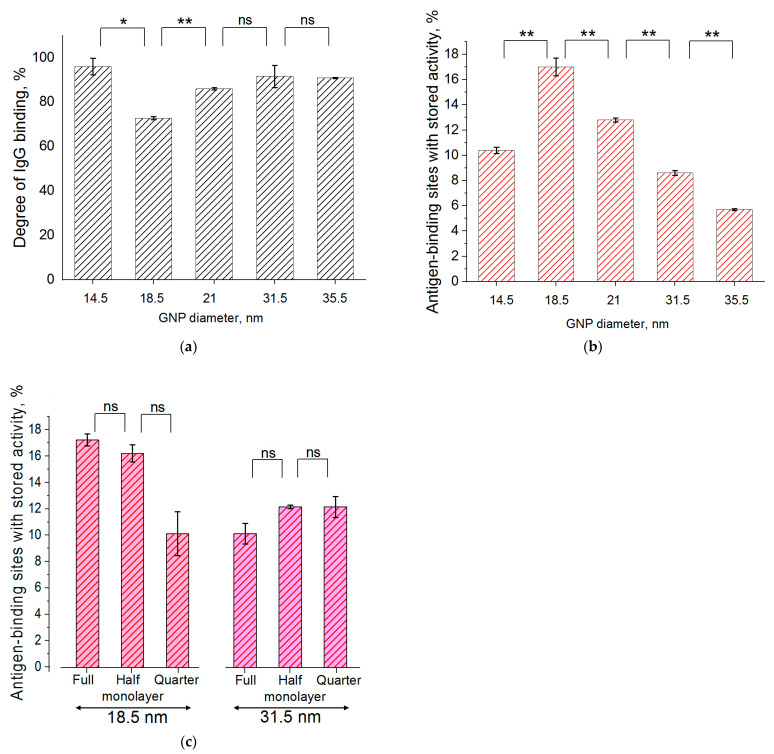
Analysis using Student’s *t*-test for (**a**) degree of antibody binding with GNPs of different diameters under monolayer binding conditions (Table 3); (**b**) percentage of stored activity for antigen-binding sites of IgG–GNP conjugates with different diameters of GNPs (Table 4), and (**c**) percentage of stored activity for antigen-binding sites of IgG–GNP conjugates with different coverages of GNP surface (Table 7). Results of the pairwise comparisons of the closest variants in the series are indicated as ns for non-significant, * for significant at *p* < 0.05, and ** for significant at *p* < 0.01.

As can be seen, the majority of the added antibodies bound to the GNPs in all preparations. The percentage of IgG binding varied from 73 to 96%, thus confirming the similarity of the conditions chosen for IgG immobilization on the surface of all five GNP preparations. Statistical analysis showed that, in this row, the neighboring preparations were significantly different only for the conjugate with an average GNP diameter equal to 18.5 nm. See Appendix B, Figure A2, for the estimation of *p* values using Student’s *t*-test, which confirms the statistical significance of the differences between pairwise-compared values (GNP1 vs. GNP2 and GNP2 vs. GNP3).

### 2.3. Determination of Active Centers of the Immobilized Antibodies (Reaction with Carboxyfluorescein)

To correctly assess the changes in the antigen-binding properties of the antibodies caused by their immobilization, we took into account the results of our earlier characterization of homogeneous interactions for the chosen fluorescein–(antibody against the fluorescein) pair [16]. The experiments conducted with the registration of fluorescence quenching in immune complexes showed that in the antibody preparation, 60% of the antibody valences were able to bind to fluorescein, and 40% of them were inactive. This value is comparable to the results of other studies for monoclonal antibodies (for example, a 1.31–1.71 stoichiometry for antigen complexes with bivalent antibodies at a theoretical 2:1 stoichiometry in [26]) and may be associated with deviations from the native structure during both the biosynthesis and storage of the antibodies. So, IgG molecules that were initially inactive (not bound to fluorescein in solution) were excluded from the consideration of immobilization-caused inactivation.

The measurement of the binding of a carboxylated fluorescein derivative to immobilized antibodies was based on the detection of the fluorescence of (i) solutions of fluorescein with known concentrations that were added for binding and (ii) a supernatant in which unbound antigen was separated from the formed immune complexes. To unify the conditions used for the measurements, the supernatants of the same precipitated GNPs were used to prepare calibration solutions of fluorescein [24].

**Figure 2:**
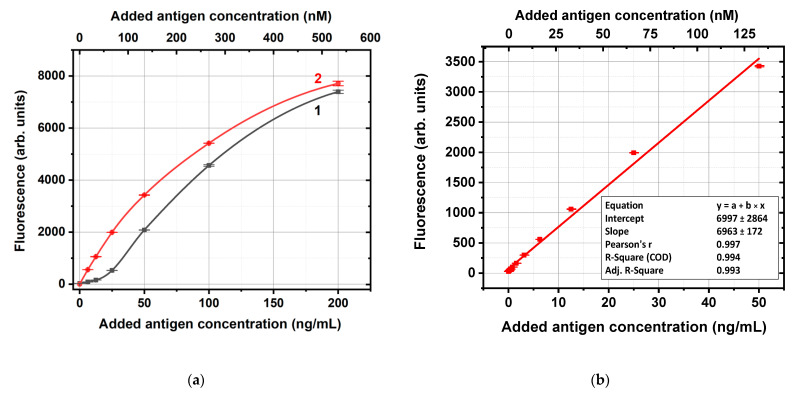
(**a**) Fluorescence values for preparations of carboxyfluorescein molecules that were not bound with the conjugate of antifluorescein IgG and GNPs (concentration of IgG in the conjugate solution—12.88 μg/mL, average diameter of GNPs—14 nm) (1) and for solutions of carboxyfluorescein in supernatants of the same GNPs (2). (**b**) Linear approximation for curve (2) in a range of carboxyfluorescein concentrations from 0 to 50 ng/mL.

Figure 2a shows, as an example, the fluorescence levels obtained using the conjugate of antifluorescein IgG and GNPs with an average diameter of 14 nm. The concentration dependence for solutions of carboxyfluorescein in supernatants in the range of concentrations from 0 to 50 ng/mL was almost linear (R^2^ > 0.99, see Figure 2b), whereas for higher concentrations, it deviated from linearity. So, the fluorescence values for supernatants with unbound carboxyfluorescein molecules (Figure 2a, curve 1) and the calibration linear approximation from Figure 2b were used to calculate the carboxyfluorescein content in these supernatants as well as the bound antigen concentrations. If some supernatants samples had fluorescence outside the calibration curve, they were additionally diluted for the following calculations.

**Figure 3:**
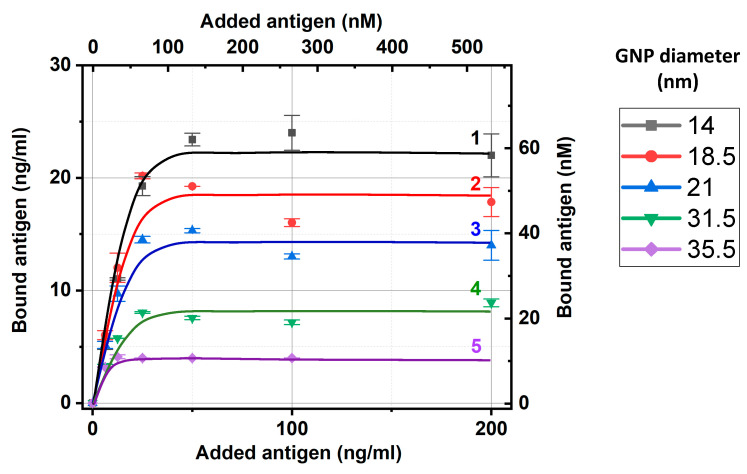
Concentrations of carboxyfluorescein bound by IgG–GNP conjugates versus concentration of added carboxyfluorescein. Dependences 1–5 align with the conjugates of GNPs with average diameters of 14, 18.5, 21, 31.5, and 35.5 nm, respectively. The experiments were implemented for the conjugate solutions with an OD of 5.0.

Considering the same experiments with all GNP preparations, we also used fluorescence for the added and nonbound carboxyfluorescein and calculated the bound carboxyfluorescein concentrations, as described above. The obtained dependences of carboxyfluorescein binding with the five tested preparations of IgG–GNP conjugates are presented in Figure 3. As can be seen, all dependencies reached saturation, which allowed the estimation of the maximal capacities of the IgG–GNP conjugates to bound antigen molecules. To unify these estimations, we used an added antigen concentration of 50 ng/mL, which aligns with the saturation regions and enabled the linear approximation of the calibration curves in the range of 0–50 ng/mL without additional dilution of the tested samples.

The relative positions of the dependences in Figure 3 reflect the differences in the surface capacity of the antibody conjugates for GNPs with different diameters. The larger the nanoparticles, the smaller their total surface area in the colloidal solution at a fixed optical density and the lower the concentration of bound antibodies per unit volume of solution. Therefore, it was logical to expect that a smaller number of antibodies would bind less carboxyfluorescein, i.e., the saturation levels in Figure 3 decrease with increasing diameter of the GNPs.

The obtained data about antigen binding at 50 ng/mL (given at Figure 3) and the conjugates’ composition (Section 2.2) were used to estimate the percentage of active antigen-binding sites in the conjugates. The results of these calculations are summarized in Table 4.

For the different-sized GNPs, the percentage of active antigen-binding sites varied more than three times, from 6% to 17%. The maximal value was reached for the GNPs with an average diameter equal to 18.5 nm; its decrease at smaller and larger diameters was statistically significant. See Appendix B, Figure A2b, for the estimation of *p* values using Student’s *t*-test for sequential pairwise comparisons. The factors that could be considered in the relationship with recorded differences included the electrostatic properties of GNPs and the differences in surface curvatures. These could influence the orientation of the antibodies, including their structural rearrangements during immobilization. Additionally, the negative surface charge of the GNPs [27], reversibly interacting with solved charged molecules, and the local charges at the surface of immobilized immunoglobulin molecules could have influenced the movement of charged antigens at this level and at the time of its stay near the binding sites of the immobilized antibodies. Of course, these interactions depend on many factors that can only be listed as subjects for further detailed studies.

The above-presented levels of the retention of antibody activity after immobilization on the surface of nanoparticles are rather low and inferior even to those of systems in which larger antigens are bound [16,25,28]. This situation requires an explanation and a search for additional factors that prevent effective binding in this case. To test the observed regularities in more detail, we performed additional experiments using another derivative of fluorescein (Section 2.4) and varying coverages of the GNP surfaces with IgG (Section 2.5).

### 2.4. Determination of Active Centers of the Immobilized Antibodies (Reaction with Aminofluorescein)

To check the impact of the electrostatic properties of the antigen derivative on the efficiency of its binding, a positively charged derivative of fluorescein containing an amino group instead of a carboxyl group was selected. Using an IgG conjugate with GNPs having an average diameter of 21 nm as an example, the antigen-binding ability of the conjugate with respect to this derivative was characterized.

**Figure 4:**
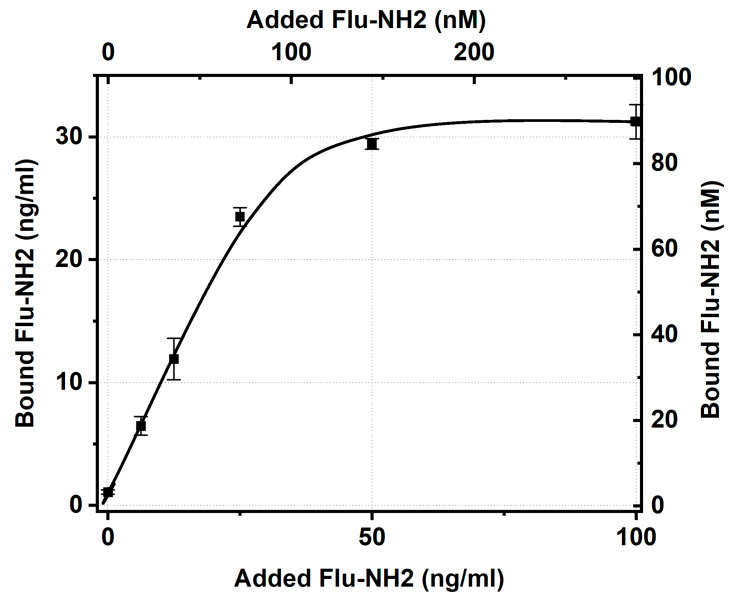
Concentrations of aminofluorescein bound by IgG–GNP conjugate (average diameter of GNPs—21 nm) versus concentration of added aminofluorescein. The experiment was conducted for a conjugate solution with an OD of 5.0.

The testing of aminofluorescein binding and the IgG–GNP conjugate reached saturation with concentration dependence (Figure 4), which allowed taking its value for an added antigen concentration of 50 ng/mL (from the plateau region, similar to the data processing for Figure 3) and calculating the percentage of antigen-binding sites of immobilized IgG with stored activity (Table 5).

As can be seen, the amino derivative of fluorescein bound to conjugated antibodies 2.1 times better than the carboxyl derivative. However, even in the case of aminofluorescein, the majority of the antibodies’ sites after immobilization lost their ability to bind this antigen. The inability to bind fluorescein, regardless of its variant, may be explained by either the complete blockage of the antigen-binding site as a result of immobilization or the structural disruption of this site caused by the interaction of IgG with the surface of the GNPs. A more complex question is why amino and carboxy derivatives, which both come into contact with the antigen-binding sites of the immobilized antibodies, differ in the limits of their binding. Probably, the antigen derivative and GNPs having the same charge limits the time required for the antigen to migrate at the GNP surface or the time needed for the transformation of the initial contact with IgG to a strong immune complex. However, the assessment of these factors’ contribution requires additional investigation.

The study results prove that the degree of antigen-binding ability stored by antibodies after their immobilization on the GNP surface is not a universal parameter for all antibodies: it depends on which antigen these antibodies bind.

### 2.5. Study of Antigen (Carboxyfluorescein)-Binding Properties of the Immobilized Antibodies with Varying Contents on the Nanoparticle Surfaces

Earlier studies of the interactions between IgG–GNP conjugates and protein antigens (peroxidase, C-reactive protein) [16,25,28] showed that the antigen-binding efficiency is sensitive to the degree of GNP coverage by the antibodies. With a decrease in the surface density of immobilization, on the one hand, the accessibility of differently located antigen-binding sites increases, and the steric hindrances for nearly located antibodies decrease. On the other hand, different degrees of coverage can affect the contacts of antibodies with the GNP surface, their orientation, and structural changes. Therefore, it was of interest to evaluate how the degree of coverage and antigen-binding properties are related in the case of antibodies and low-molecular-weight antigens.

Using GNPs with average diameters of 18.5 and 31.5 nm, conjugates were synthesized containing different amounts of antibodies on the surface, namely, (i) with a completely filled monolayer of antibodies, (ii) containing two times fewer antibodies, and (iii) containing four times fewer antibodies. Firstly, they were characterized in terms of IgG binding. As can be seen from Table 6, all preparations demonstrated high percentages of binding that were comparable with the earlier considered (Table 3) monolayer preparations.

**Figure 5:**
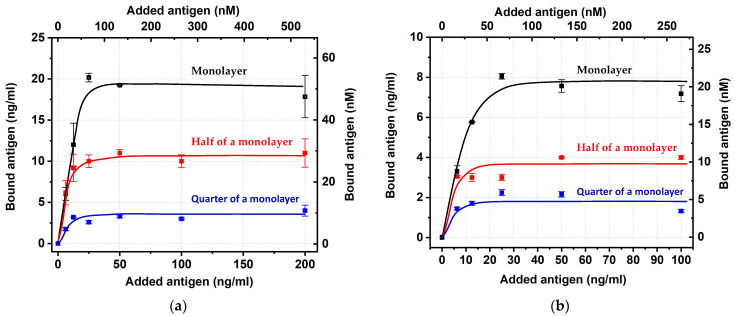
Dependence of the concentrations of carboxyfluorescein bound by IgG–GNP conjugates on the initially added carboxyfluorescein for 18.5 nm (**a**) and 31.5 nm (**b**) GNP conjugates with different antibody contents in the conjugates. Dependences correspond to one monolayer (black) and half (red) and one-quarter of a monolayer (blue) of IgG.

Next, the conjugates were tested for binding with carboxyfluorescein. The obtained concentration dependences (Figure 5) reached saturation in all six cases, thus allowing the estimation of the percentage of the antigen-binding sites with stored activity. The calculations were implemented basing on the binding values for an added antigen concentration equal to 50 ng/mL (similar to the data processing for Figure 3 and Figure 4), which, in all cases, agreed with the plateau regions.

The calculated percentage values did not reveal any patterns regarding the degree of coverage (see Table 7 and Figure A2c in Appendix B). Thus, in the situation with the absence of the above-listed factors that influence the interactions with protein antigens, the preparations of IgG–GNP conjugates with different IgG contents were comparable in their reactivity for the binding of low-molecular-weight antigens. Additionally, the IgG:GNP ratio is a useful tool for modulating the analytical parameters of immunosensors [29,30,31,32]; the obtained data confirmed the possibility of applying these tools without risks caused by antibody immobilization.

## 3. Materials and Methods

### 3.1. Immunoreactants

**Figure 6:**
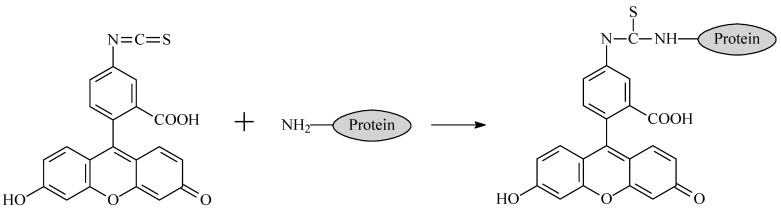
Conjugation of BSA with fluorescein isothiocyanate.

**Figure 7:**
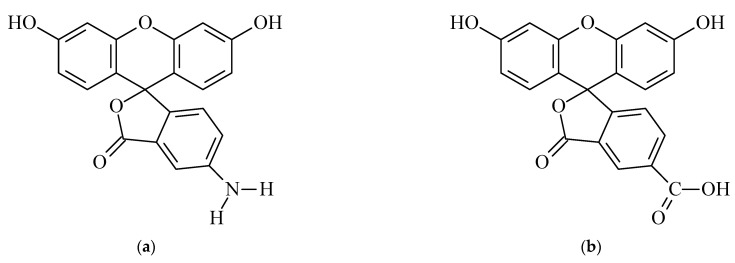
Structures of 5-aminofluorescein (**a**) and 5-carboxyfluorescein (**b**).

Mouse monoclonal antibodies against fluorescein (cat. No 5F3cc, isotype IgG1, clone 2A3cc, IgG1, purification using protein A Sepharose, storage formulation—solution in phosphate buffer saline, pH 7.4, with 0.09% NaN_3_) were obtained from HyTest (Moscow, Russia). The immunogen used to obtain them was a conjugate of bovine serum albumin (BSA) with fluorescein isothiocyanate see Figure 6. The used fluorescein variants included 5-carboxyfluorescein and 5-aminofluorescein from Lumiprobe (Moscow, Russia) (Figure 7).

### 3.2. Other Materials

Gold (III) chloride, poly(vinyl formal), Tris, and sodium citrate were obtain from Sigma-Aldrich (St. Louis, MO, USA). Poly(ethylene glycol) with a reactive free thiol (PEG-SH; MW 5 kDa) was from Creative PEGWorks (Durham, NC, USA). All salts, acids, and other additional reactants were obtained from Chimmed (Moscow, Russia). To prepare buffer solutions, deionized water was obtained using a Milli-Q system (Millipore; Burlington, MA, USA).

### 3.3. Synthesis of GNPs

Five preparations of spherical GNPs were synthesized via the Frens technique of HAuCl_4_ reduction [33]. Concentrations of reagents for reaching various average diameters of the GNPs previously were selected in [16]. Briefly, 1% HAuCl_4_ solution was added to deionized water and brought to a boil, and 1% sodium citrate was added under stirring. For the five syntheses, the sodium citrate solution volumes were 2.5, 1.88, 1.75, 1.5, and 1.2 mL; the HAuCl_4_ solution volume was 1.0 mL; and water was added to a volume of 100 mL. The reaction mixture was boiled for 25 min, cooled, and stored at 4–6 °C. The obtained preparations were designated GNP1–GNP5, respectively.

### 3.4. Transmission Electron Microscopy of GNPs

The GNP preparations were applied to nets (300 mesh) that were covered with a poly(vinyl formal) film dissolved in chloroform. The images were obtained with a transmission electron microscope (JEM CX-100, Jeol; Tokyo, Japan) at an accelerating voltage of 80 kV and a magnification of ×3,300,000. The digital photographs were analyzed using Image Tool 3.0 software (University of Texas Health Science Center, San Antonio, TX, USA). The obtained average diameters of the GNPs were used to calculate the nanoparticle number per mL in their colloidal solution based on their near-spherical shape, the density of gold, and the complete reduction of gold salt in the course of the used synthetic technique (see Section 3.3).

### 3.5. Spectroscopy of GNP Solutions

The absorption of the obtained colloidal solutions of GNPs was recorded in the wavelength range of 400–700 nm using a spectrophotometer (Libra S80, Biochrom; Cambridge, UK).

### 3.6. Obtaining Antibody–GNP Conjugates with Different Compositions

Using the synthesized GNP preparations, conjugates with the anti-fluorescein IgG were obtained via adsorption immobilization. Before the conjugation, IgG was dialyzed for 2 h at 4 °C vs. a 1000-fold excess of 10 mM Tris-HCl buffer, pH 9.0. Then, 0.1 M K_2_CO_3_ was added to the GNP solutions (diluted to an OD = 1.0 at the peak wavelength) until a pH of 9.0 was reached. After this, the GNPs and the IgG solutions were mixed and incubated for 30 min at 20–22 °C under stirring. Then, an aqueous solution of PEG-SH was added to a final concentration of 0.25%. After 10 min, the obtained IgG–GNP conjugates were purified via precipitation using an Allegra 64R centrifuge (Beckman Coulter; Indianapolis, IN, USA). The centrifugation was implemented for 15 min at 4 °C under 27,000× *g*, 25,000× *g*, 23,000× *g*, 20,000× *g*, and 17,800× *g* for IgG conjugates with GNP1–5, respectively. (The same centrifugation was also implemented for GNP1–5 preparations without adding IgG to obtain the diluted solutions for the experiments described in Section 3.7) The pellets were resuspended in 50 mM phosphate buffer with 100 mM NaCl and 0.25% PEG-SH, diluting the obtained antibody–GNP conjugates to an OD = 5.0. The conjugates were stored at 4–6 °C. The stabilizing effect of the PEG-SH addition is caused by the known strong affinity of SH groups to the surface of gold [34]. This treatment excluded self-aggregation of the stored antibody–GNP conjugates and did not cause nonspecific interactions, as confirmed for the GNP conjugates with antibodies of other specificities that were stabilized using the same protocol.

Moreover, the tested GNP conjugates with antibodies of other specificities and PEG-SH-based stabilization demonstrated the absence of any interaction with the antigen.

### 3.7. Measurements of Antibodies Bound to GNPs

The supernatants after the centrifugation of native GNP1–5 (see Section 3.6) were used to dilute antifluorescein antibodies. The tryptophan fluorescence of the obtained preparations (excitation wavelength 280 nm, emission wavelength 350 nm) was measured using EnSpire 2300 microplate reader (Perkin Elmer, Waltham, MA, USA). A linear approximation of the obtained concentration dependence was used as the calibration curve.

The tryptophan fluorescence of the supernatants obtained after the second centrifugation of IgG conjugates for GNP1–5 (see Section 3.6) was measured under the same excitation and emission wavelengths and compared with the calibration curve to calculate the concentration of nonbound IgG. The content of IgG in the complexes formed with GNP1–5 was calculated as the difference between the concentration of IgG in the solution initially added for the conjugation and the concentration of unbound IgG in the obtained supernatant [16].

### 3.8. Measurements of Active Antibodies on the GNP Surface

The obtained conjugates of GNPs with different diameters and the antifluorescein IgG were diluted to an OD of 5.0 and separated into seven aliquots. Fluorescein was added to six aliquots to reach concentrations of 200, 100, 50, 25, 12.5, and 6.25 ng/mL, and the seventh aliquot was used without additions. The preparations were incubated for 30 min at room temperature under stirring and then centrifuged twice following the aforementioned regimes (see Section 3.6). The supernatants from the second centrifugation were used to determine the contents of bound and unbound fluorescein in the same way as described above (see Section 3.7) for bound and unbound antibodies [16]. The fluorescence of fluorescein was registered under an excitation wavelength of 501 nm and an emission wavelength of 522 nm. An EnSpire 2300 microplate reader from Perkin Elmer (Waltham, MA, USA) and white microplates from Nunc (Roskilde, Denmark) were used for these measurements.

## 4. Conclusions

Despite the small size of the studied antigen fluorescein, only a minority (no more than 27%) of the binding sites for IgG on the surface of gold nanoparticles were able to interact with the antigen after immobilization. These results demonstrate that the previously discovered inactivation of antibodies in complexes with gold nanoparticles cannot be explained by steric limitations alone.

The obtained data indicate the influence of the size of the nanoparticles on the retention of the activity of immobilized antibodies. The maximum degree of retained antigen-binding properties was observed for the conjugate with gold nanoparticles that had an average diameter of 18.5 nm, which decreased with both increases and decreases in the nanoparticle size.

An important new result is the influence of the charge of the hapten outside its antibody-binding part on the ability to interact with immobilized antibodies. Charged groups, not excluding the contact of an antibody–nanoparticle conjugate with an antigen, can significantly change the number of immobilized antibody sites with which immune complexes can be formed with a given antigen derivative.

It should also be noted that in the case of a low-molecular-weight antigen, a decrease in the surface density of immobilized antibodies cannot be considered as a tool that ensures greater efficiency antigen binding. In the experiments performed, monolayer immobilization did not reliably reduce the percentage of reactive binding sites of the antibodies.

Of course, the mechanisms of antibodies’ inactivation in the course of their immobilization require further detailed studies, with direct characterization of the orientation, contacts, and structural changes of the immunoglobulins on the surfaces of nanoparticles. However, the importance of the data obtained in this work lies in expanding the list of factors that should be taken into account and explained using the integral concept of structural–functional relationships. The prediction of antibodies’ properties on the surface of nanoparticles will allow the targeted development of effective immunosensors instead of through empirical and labor-intensive experimental comparisons of different preparations.

## Figures and Tables

**Table 1:** Microscopic and spectral characterization of GNPs.

Code of GNP Preparation in Methods	GNP Diameter (TEM Data), nm	Ellipticity	Peak of Absorption Spectrum, nm	GNP Diameter (Spectral Data), nm
GNP1	13.9 ± 2.2	1.20 ± 0.14	517.5 ± 1.0	13 ± 1.3
GNP2	18.4 ± 0.7	1.12 ± 0.13	520.2 ± 0.2	18.5 ± 0.3
GNP3	21.3 ± 2.3	1.08 ± 0.04	521.1 ± 0.1	21 ± 0.2
GNP4	31.5 ± 5.3	1.19 ± 0.14	524.5 ± 0.1	30 ± 0.1
GNP5	35.5 ± 8.0	1.28 ± 0.41	526.7 ± 0.3	36 ± 0.4

Values are reported as mean ± SD.

**Table 2:** Calculation of reactant concentrations for monolayer immobilization of antibodies on surface of GNP preparations.

GNP Diameter, nm	GNP, Individual Surface Area, nm^2^	GNP in the Reaction Mixture, OD	GNP, Particles per mL	GNP, Total Surface Area per mL, nm^2^	IgG for Monolayer Coverage, Molecules per mL	IgG in the Reaction Mixture, μg/mL
14	530	0.861	2.3 × 10^12^	12.1 × 10^14^	5.4 × 10^13^	13.42
18.5	1075	0.913	7.8 × 10^11^	8.3 × 10^14^	3.8 × 10^13^	9.44
21	1385	0.901	5.3 × 10^11^	7.3 × 10^14^	3.3 × 10^13^	8.32
31.5	2826	0.910	1.8 × 10^11^	5.0 × 10^14^	2.3 × 10^13^	5.82
35.5	4069	0.943	1.1 × 10^11^	4.5 × 10^14^	1.9 × 10^13^	4.85

**Table 3:** Antibodies binding with GNPs under the chosen conditions for monolayer immobilization.

GNP Diameter, nm	GNP, Particles per mL	IgG in the Reaction Mixture, μg/mL	Unbound IgG, μg/mL	Bound IgG per mL, μg	Average Degree of IgG Binding, %	Composition (IgG: GNP) of the Conjugates
14	2.3 × 10^12^	13.42	0.54 ± 0.06	12.88 ± 0.06	96	22:1
18.5	7.8 × 10^11^	9.44	2.56 ± 0.07	6.88 ± 0.07	73	35:1
21	5.3 × 10^11^	8.32	1.18 ± 0.04	7.14 ± 0.04	86	54:1
31.5	1.8 × 10^11^	5.82	0.49 ± 0.01	5.33 ± 0.01	92	119:1
35.5	1.1 × 10^11^	4.85	0.44 ± 0.03	4.41 ± 0.03	91	161:1

**Table 4:** Antigen-binding capacities of the IgG-GNP conjugates (studies with carboxyfluorescein).

GNP Diameter, nm	GNP after Concentration, OD	GNP, Particles per mL	Concentration of Antigen-Binding Sites *, nM	Concentration of Bound Antigen, nM	Average Percentage of Antigen-Binding Sites with Stored Activity, %
14	5	1.33 × 10^13^	598 ± 36	62 ± 4.0	10
18.5	5	4.27 × 10^12^	301 ± 21	51 ± 0.7	17
21	5	2.94 × 10^12^	317 ± 13	41 ± 0.5	13
31.5	5	9.89 × 10^11^	235 ± 2	20 ± 0.8	9
35.5	5	5.83 × 10^11^	186 ± 6	11 ± 0.3	6

Quantities of antigen-binding sites were calculated here and in the tables bellow based on the bivalence of IgG molecules and the exclusion of the nonactive (in homogeneous testing) IgG fraction [16]. The RSD values for measurements of unbound IgG concentrations were not more than 12%. * Taking into account 60% of the active valences of antibodies in the original preparation.

**Table 5:** Antigen-binding capacity of IgG–GNP conjugate (average diameter of GNPs—21 nm; study with aminoxyfluorescein).

GNP Diameter, nm	GNP after Concentration, OD	GNP, Particles per mL	Concentration of Antigen-Binding Sites, nM	Concentration of Bound Antigen, nM	Average Percentage of Antigen-Binding Sites with Stored Activity, %
21	5	2.94 × 10^12^	333 ± 25	90 ± 4	27

**Table 6:** Antibodies binding with GNPs under immobilization with different degree of coverage.

Diameter of GNP, nm	Degree of Coverage	Concentration of Added IgG, μg/mL	Concentration of Non-Bound IgG, μg/mL	Concentration of Bound IgG, μg/mL	Average Percentage of IgG Binding, %
18.5	Full monolayer	9.44	2.56 ± 0.07	6.88 ± 0.07	73
18.5	Half of monolayer	4.72	0.44 ± 0.19	4.27 ± 0.19	91
18.5	Quarter of monolayer	2.36	0.33 ± 0.14	2.03 ± 0.14	86
31.5	Full monolayer	5.82	0.49 ± 0.08	5.33 ± 0.08	92
31.5	Half of monolayer	2.91	0.56 ± 0.10	2.34 ± 0.10	81
31.5	Quarter of monolayer	1.45	0.27 ± 0.19	1.19 ± 0.19	82

**Table 7:** Antigen-binding activity of immobilized antibodies for different coverages of the GNP surface.

GNP Diameter, nm	Degree of Coverage	GNP after Concentration, OD	GNP, Particles per mL	Concentration of Antigen-Binding Sites, nM	Concentration of Bound Antigen, nM	Average Percentage of Antigen-Binding Sites with Stored Activity, %
18.5	Full monolayer	5	7.8 × 10^11^	301 ± 3	54 ± 1.4	17
18.5	Half of monolayer	5	7.8 × 10^11^	187 ± 8	29 ± 1.2	16
18.5	Quarter of monolayer	5	7.8 × 10^11^	89 ± 6	11 ± 1.8	10
31.5	Full monolayer	5	1.8 × 10^11^	235 ± 4	24 ± 1.8	10
31.5	Half of monolayer	5	1.8 × 10^11^	103 ± 4	11 ± 0.3	10
31.5	Quarter of monolayer	5	1.8 × 10^11^	52 ± 8	6 ± 0.4	11

## Data Availability

The data that support the findings of this study are available from the corresponding author upon request.

## References

[1] Zhu G., Yin X., Jin D., Zhang B., Gu Y., An Y. (2019). Paper-based immunosensors: Current trends in the types and applied detection techniques. TrAC—Trends Anal. Chem..

[2] Meng X., O’Hare D., Ladame S. (2023). Surface immobilization strategies for the development of electrochemical nucleic acid sensors. Biosens. Bioelectron..

[3] Liu Y., Yu J. (2016). Oriented immobilization of proteins on solid supports for use in biosensors and biochips: A review. Microchim. Acta.

[4] Iijima M., Kuroda S. (2017). Scaffolds for oriented and close-packed immobilization of immunoglobulins. Biosens. Bioelectron..

[5] Shen M., Rusling J.F., Dixit C.K. (2017). Site-selective orientated immobilization of antibodies and conjugates for immunodiagnostics development. Methods.

[6] Welch N.G., Scoble J.A., Muir B.W., Pigram P.J. (2017). Orientation and characterization of immobilized antibodies for improved immunoassays (Review). Biointerphases.

[7] Park M. (2019). Orientation control of the molecular recognition layer for improved sensitivity: A review. BioChip J..

[8] Gan S.Y., Tye G.J., Chew A.L., Ng W.K., Lai N.S. (2023). Linker-mediated oriented antibody immobilisation strategies for a more efficient immunosensor and diagnostic applications: A review. Biosens. Bioelectron..

[9] Farka Z., Juřík T., Kovář D., Trnková L., Skládal P. (2017). Nanoparticle-based immunochemical biosensors and assays: Recent advances and challenges. Chem. Rev..

[10] Zhang L., Mazouzi Y., Salmain M., Liedberg B., Boujday S. (2020). Antibody-gold nanoparticle bioconjugates for biosensors: Synthesis, characterization and selected applications. Biosens. Bioelectron..

[11] Nguyen V.-T., Song S., Park S., Joo C. (2020). Recent advances in high-sensitivity detection methods for paper-based lateral-flow assay. Biosens. Bioelectron..

[12] Di Nardo F., Chiarello M., Cavalera S., Baggiani C., Anfossi L. (2021). Ten years of lateral flow immunoassay technique applications: Trends, challenges and future perspectives. Sensors.

[13] Sotnikov D.V., Zherdev A.V., Dzantiev B.B. (2015). Development and application of a label-free fluorescence method for determining the composition of gold nanoparticle—protein conjugates. Intern. J. Mol. Sci..

[14] Ruiz G., Tripathi K., Okyem S., Driskell J.D. (2019). pH impacts the orientation of antibody adsorbed onto gold nanoparticles. Bioconjug. Chem..

[15] Filbrun S.L., Driskell J.D. (2016). A fluorescence-based method to directly quantify antibodies immobilized on gold nanoparticles. Analyst.

[16] Sotnikov D.V., Byzova N.A., Zherdev A.V., Dzantiev B.B. (2021). Retention of activity by antibodies immobilized on gold nanoparticles of different sizes: Fluorometric method of determination and comparative evaluation. Nanomaterials.

[17] Sotnikov D.V., Byzova N.A., Zherdev A.V., Dzantiev B.B. (2023). Changes in antigen-binding ability of antibodies caused by immobilization on gold nanoparticles: A case study for monoclonal antibodies to fluorescein. Biointer. Res. Appl. Chem..

[18] Rahman M., Laurent S., Tawil N., Yahia L., Mahmoudi M. (2013). Protein-Nanoparticle Interactions.

[19] Sotnikov D.V., Berlina A.N., Ivanov V.S., Zherdev A.V., Dzantiev B.B. (2019). Adsorption of proteins on gold nanoparticles: One or more layers?. Coll. Sur. B Biointerfaces.

[20] Mishra A., Das P.K. (2022). Thermodynamics of multilayer protein adsorption on a gold nanoparticle surface. Phys. Chem. Chem. Phys..

[21] Monopoli M.P., Walczyk D., Campbell A., Elia G., Lynch I., Baldelli Bombelli F., Dawson K.A. (2011). Physical—chemical aspects of protein corona: Relevance to in vitro and in vivo biological impacts of nanoparticles. J. Amer. Chem. Soc..

[22] Liu W., Rose J., Plantevin S., Auffan M., Bottero J.Y., Vidaud C. (2013). Protein corona formation for nanomaterials and proteins of a similar size: Hard or soft corona?. Nanoscale.

[23] Docter D., Westmeier D., Markiewicz M., Stolte S., Knauer S.K., Stauber R.H. (2015). The nanoparticle biomolecule corona: Lessons learned—challenge accepted?. Chem. Soc. Rev..

[24] Makaraviciute A., Ramanaviciene A. (2013). Site-directed antibody immobilization techniques for immunosensors. Biosens. Bioelectron..

[25] Tripathi K., Driskell J.D. (2018). Quantifying bound and active antibodies conjugated to gold nanoparticles: A comprehensive and robust approach to evaluate immobilization chemistry. ACS Omega.

[26] Reader P.P., Olkhov R.V., Reeksting S., Lubben A., Hyde C.J., Shaw A.M. (2019). A rapid and quantitative technique for assessing IgG monomeric purity, calibrated with the NISTmAb reference material. Anal. Bioanal. Chem..

[27] Mays D.C. (2007). Using the Quirk-Schofield diagram to explain environmental colloid dispersion phenomena. J. Nat. Res. Life Sci. Educ..

[28] Zhang L., Hu D., Salmain M., Liedberg B., Boujday S. (2019). Direct quantification of surface coverage of antibody in IgG-Gold nanoparticles conjugates. Talanta.

[29] Zvereva E.A., Byzova N.A., Sveshnikov P.G., Zherdev A.V., Dzantiev B.B. (2015). Cut-off on demand: Adjustment of the threshold level of an immunochromatographic assay for chloramphenicol. Anal. Methods.

[30] Zhang X., Fishlock S., Sharpe P., McLaughlin J. (2022). Development of colorimetric lateral flow assays with gold nanostructures for cystatin C detection. Sens. Act. Rep..

[31] Cavalera S., Pezzoni G., Grazioli S., Brocchi E., Baselli S., Lelli D., Colitti B., Serra T., Di Nardo F., Chiarello M. (2022). Investigation of the “antigen hook effect” in lateral flow sandwich immunoassay: The case of lumpy skin disease virus detection. Biosensors.

[32] Wu P., Prachyathipsakul T., Huynh U., Qiu J., Jerry D.J., Thayumanavan S. (2023). Optimizing conjugation chemistry, antibody conjugation site, and surface density in antibody-nanogel conjugates (ANCs) for cell-specific drug delivery. Bioconjug. Chem..

[33] Frens G. (1973). Controlled nucleation for the regulation of the particle size in monodisperse gold suspensions. Nature.

[34] Chatterjee S., Lou X.Y., Liang F., Yang Y.W. (2022). Surface-functionalized gold and silver nanoparticles for colorimetric and fluorescent sensing of metal ions and biomolecules. Coord. Chem. Rev..

